# Improved Visualization of Prostate Cancer Using Multichannel Computed Diffusion Images: Combining ADC and DWI

**DOI:** 10.3390/diagnostics12071592

**Published:** 2022-06-30

**Authors:** Matthias Hammon, Marc Saake, Frederik B. Laun, Rafael Heiss, Nicola Seuss, Rolf Janka, Alexander Cavallaro, Michael Uder, Hannes Seuss

**Affiliations:** 1 Department of Radiology, University Hospital Erlangen, Friedrich-Alexander-Universität Erlangen-Nürnberg, Maximiliansplatz 3, 91054 Erlangen, Germany; matthias.hammon@gmail.com (M.H.); marc.saake@uk-erlangen.de (M.S.); frederik.laun@uk-erlangen.de (F.B.L.); rafael.heiss@uk-erlangen.de (R.H.); rolf.janka@uk-erlangen.de (R.J.); alexander.cavallaro@uk-erlangen.de (A.C.); michael.uder@uk-erlangen.de (M.U.); 2 Institute of Neuroradiology and Radiology, Klinikum Fürth, Jakob-Henle-Str. 1, 90766 Fürth, Germany; nlk246@hotmail.com; 3 Department of Radiology, Klinikum Forchheim, Krankenhausstrasse 10, 91301 Forchheim, Germany

**Keywords:** MR diffusion/perfusion, prostate, primary neoplasms, image manipulation/reconstruction, general computer applications, observer performance

## Abstract

(1) Background: For the peripheral zone of the prostate, diffusion weighted imaging (DWI) is the most important MRI technique; however, a high b-value image (hbDWI) must always be evaluated in conjunction with an apparent diffusion coefficient (ADC) map. We aimed to unify the important contrast features of both a hbDWI and ADC in one single image, termed multichannel computed diffusion images (mcDI), and evaluate the values of these images in a retrospective clinical study; (2) Methods: Based on the 2D histograms of hbDWI and ADC images of 70 patients with histologically proven prostate cancer (PCa) in the peripheral zone, an algorithm was designed to generate the mcDI. Then, three radiologists evaluated the data of 56 other patients twice in three settings (T2w images +): (1) hbDWI and ADC; (2) mcDI; and (3) mcDI, hbDWI, and ADC. The sensitivity, specificity, and inter-reader variability were evaluated; (3) Results: The overall sensitivity/specificity were 0.91/0.78 (hbDWI + ADC), 0.85/0.88 (mcDI), and 0.97/0.88 (mcDI + hbDWI + ADC). The kappa-values for the inter-reader variability were 0.732 (hbDWI + ADC), 0.800 (mcDI), and 0.853 (mcDI + hbDWI + ADC). (4) Conclusions: By using mcDI, the specificity of the MRI detection of PCa was increased at the expense of the sensitivity. By combining the conventional diffusion data with the mcDI data, both the sensitivity and specificity were improved.

## 1. Introduction

Prostate cancer (PCa) is the most frequently occurring type of cancer in US American men and accounts for roughly one in five cancers [1]. Multiparametric magnetic resonance imaging (mpMRI) of the prostate is an established noninvasive alternative to a transrectal ultrasound-guided prostate biopsy (TRUS biopsy) to rule out clinically significant cancers [2]. This examination is highly standardized by PI-RADS v 2.1 [3]. In the peripheral zone (PZ), diffusion weighted images (DWI) and the apparent diffusion coefficient map (ADC) calculated are defined as the dominant contrast mechanism for the detection and characterization of lesions [3,4]. The cell walls of the densely packed tumor cells and the decreased extracellular space function as barriers to the diffusion, which results in a bright signal in high b-value DWI (hbDWI). However, not all bright lesions are a true diffusion restriction. Therefore, is necessary to acquire the images with different strengths of diffusion weighting (b-values) and calculate their differences with an ADC. Suspicious lesions have a high intensity in hbDWI and a low ADC value. This combination is important, because some lesions exhibit a high signal both in hbDWI and in ADC (T2 shine-through effect), and some have a low signal in both images (blackout) [5,6].

Since 75–80% of PCa cases occur in the peripheral zone, excellent acquisition and a thorough evaluation of the DWI and ADC are of paramount importance. It should be noted that PI-RADS v2.1 recommends the inclusion of hbDWI but leaves to the investigator’s discretion whether images with high b-values (e.g., 1500 s/mm^2^) are actually measured or computed from images acquired with lower b-values (typically below 1000 s/mm^2^), resulting in the generation of computed diffusion weighted images (cDWI) [7,8,9,10]. A radiological evaluation is then performed by reading the hbDWI and ADC.

It is not uncommon to merge the information from several channels into one image. For example, on a monitor, the red, green, and blue channels are combined to create a color image. We hypothesized that combining the information from the two channels hbDWI and ADC into a single image series could facilitate prostate MRI reporting. Inspired by the success of the cDWI approach, we sought to incorporate both the hbDWI and ADC into one grayscale multichannel computed diffusion image (mcDI) for prostate imaging; to our knowledge, this has not been done before.

The aims of this study were to first analyze the hbDWI/ADC properties of normal prostate tissue and PCa with a two-dimensional (2D) histogram, second, to use this information to develop an algorithm for calculating such images and, finally, to evaluate the additional value of this approach in a clinical study.

## 2. Materials and Methods

This retrospective study was conducted in accordance with the guidelines of the Declaration of Helsinki, and this retrospective study was approved by the Ethics Committee of the University Hospital Erlangen. The Ethics Committee waived the written informed consent requirement.

### 2.1. Patients

Fifty-six consecutive male patients (mean age: 68 years (range: 51–84 years)) who had received an mpMRI of the prostate between January and April 2019 due to an elevated PSA serum level were included. Patients were included in the cancer group if at least one PI-RADS 4 or 5 lesion in the peripheral zone was reported in the clinical MRI report, and a TRUS biopsy within 3 months confirmed a significant (Gleason score ≥ 7) PCa in that location. Patients were included in the control group if they had a PI-RADS score of 1 or 2 and received a systematic biopsy within 3 months that did not yield a clinically significant cancer. The exclusion criteria were a negative MRI (PI-RADS 1 or 2) in combination with a positive biopsy, a positive MRI (PI-RADS 4 or 5) in combination with a negative biopsy, an indecisive MRI (PI-RADS 3), a suspicious lesion or proven cancer only in the central or transitional zone (TZ), and nondiagnostic images, e.g., due to the presence of a hip replacement prosthesis. Another 70 MRI datasets from patients (mean age: 67 years (range: 52–85 years)) with histologically proven PCa were used as a training set to create the histograms as described below.

### 2.2. Image Acquisition

All MRI examinations had been performed during clinical routine examinations on one of two 3 Tesla clinical magnetic resonance scanners (Magnetom Skyra fit/Magnetom Vida, Siemens Healthcare GmbH, Erlangen, Germany) with an 18-channel body coil and a 32-channel spine array. We included T2-weighted images in transverse orientation using a turbo–spin–echo sequence (settings: time of repetition (TR) 4000/7480 ms, time of echo (TE) 101/105 ms, slice thickness (ST) 3 mm, and flip angle 150°/160°) and transverse DWI using an echo–planar–imaging sequence (settings: TR 5090/5660 ms, TE 57/59 ms, and flip angle 180°) at b0 = 0 s/mm^2^ and b800 = 800 s/mm^2^ with an in-plane resolution of 1.7 × 1.7/0.85 × 0.85 mm^2^ and an ST of 3.5 mm. From these DWI, a b1500 = 1500 s/mm^2^ dataset and an ADC were calculated based on the mono-exponential model as described by Blackledge et al. [11].

### 2.3. Image Processing

Image processing was performed in Python (versions 3.7–3.9, Python Software Foundation) and Photoshop (version CC, Adobe Inc., San Jose, CA, USA). All DWIs were normalized to the voxel, with the maximum intensity on a per-patient basis (combining all b-values and all slices). All ADCs were normalized to the potential maximum intensity of 4095 mm^2^/s.

### 2.4. Image Segmentation

Before creating the actual mcDI, we had to gather information about the behavior of tumor and prostate voxels. Therefore, two board-certified radiologists jointly performed a voxel-based image segmentation of the 70 patients in the training dataset. The ADCs were imported into Photoshop. The multiple 2D images of the stack were imported as separate layers, and each layer was segmented separately. The segmentations were itemized into PCa (red), PZ (green), and transitional/central zone (blue). The non-prostate tissue/background was kept in the original grayscale.

### 2.5. Two-Dimensional DWI-ADC Histogram

**Figure 1:**
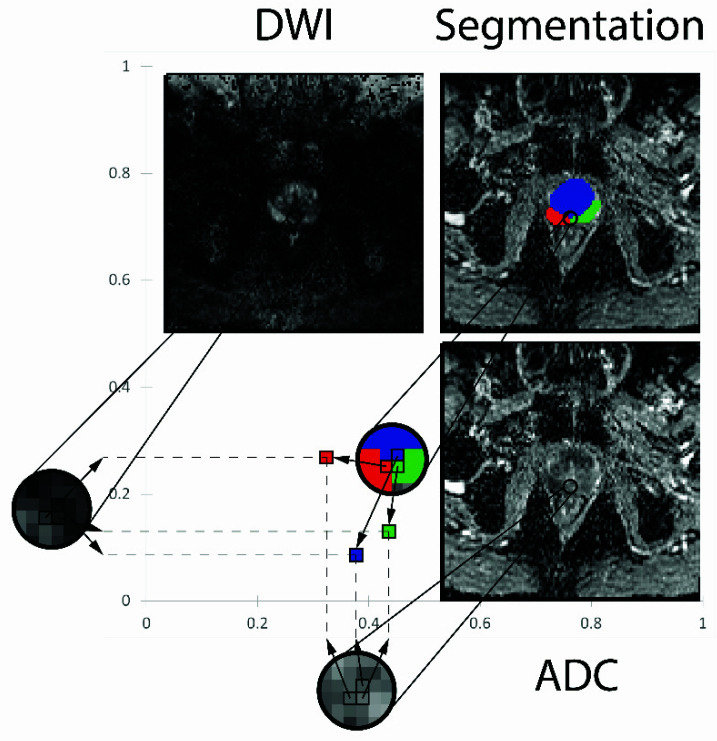
Creation of the 2D histograms. Every pixel in a two-dimensional (2D) histogram contains information from three different voxels. The normalized intensity of the DWI is plotted on the *y*-axis and the normalized ADC value on the *x*-axis. Furthermore, the images are segmented to enable differentiation of the underlying tissue and color-coding thereof in the final 2D histogram. For example, the tumor voxel has the coordinates (0.32, 0.27) and is colored in red. Additionally, two more voxels are plotted for the transitional zone (blue) and the peripheral zone (green).

To visualize the appearance of the tumor, normal prostate tissue, and background, 2D DWI-ADC histograms were computed, with 256 bins in each dimension. Each voxel in the corresponding segmented area was mapped to the x- and y-coordinates given by its normalized ADC and DWI values (Figure 1). For each voxel that was mapped to a certain point on the histogram, the value of this point was increased by one. For example, a tumor voxel with a normalized ADC value of 0.3 and a normalized DWI value of 0.2 was mapped to point A with an x-coordinate of 0.3 and a y-coordinate of 0.2. Therefore, point A can be written as A (ADC or DWI) or A (0.3 or 0.2). To increase the visibility of small values, the final intensity I of the map was defined as the integer of the natural logarithm of the value A:

(1) I= int(lnA)

The base value A of each pixel was set to 1 to yield an intensity I=0 if no voxels were mapped.

### 2.6. Multichannel Computed Diffusion Images

**Figure 2:**
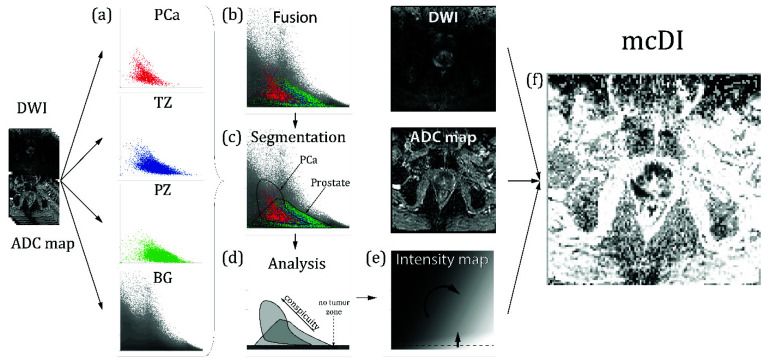
Creation of the map and the mcDI. (**a**) The ADCs of 70 patients are segmented to create four different 2D histograms (prostate cancer (PCa), red; peripheral zone (PZ), green; transitional zone (TZ), blue; and background (BG), gray). See Figure 1 for further information. It is important to note that the pixel brightness is dictated by the number of voxels plotted into one pixel; the darker the pixel, the more voxels it represents. Due to the exponential representation, every brightness step represents an exponential increase in the voxel density; (**b**) Fusion: The four 2D histograms are fused to show all the tissues in a single image (for demonstration purposes, a simple image overlay was chosen); (**c**) Segmentation: The respective areas are segmented into the tumor voxel (PCa) and prostate tissue; (**d**) Analysis: The segmented areas are analyzed. In concordance with the clinical knowledge, we found that the tumor voxel had an extensive range of DWI and ADC values. However, no tumor was found below a certain DWI threshold, which defined the ‘no-tumor zone’. Furthermore, the conspicuity of the tumor voxels increased with the increasing intensity in the DWI and decreasing intensity in the ADC; (**e**) Map: These findings were used to create a map that forms the basis of the multichannel computed diffusion images (mcDI). The no-tumor zone was implemented by suppressing the low DWI values. For that purpose, a gradient was used with decreasing brightness correlating to the increasing DWI values. We chose hypo-intensity to represent conspicuous areas, so the resulting mcDI has an ADC-like appearance. However, inverted intensity maps can also be used to generate a more DWI-like image. Since our images exhibit ADC-like characteristics, the main gradient runs vertically from black (ADC = 0) to white (ADC = 1). To include the DWI information, the gradient was tilted clockwise. It is important to notice that the increasing signal in the DWI corresponds to the decreasing intensity in the mcDI; (**f**) mcDI: The mcDIs are created by reversing the algorithm of the histograms and plotting the intensity of the corresponding location on the map back to the final image. Compared to the ADC, the signal of the fatty tissue and bones is suppressed (lighter), while the tumor tissue is even darker than in the original ADC. The effect on the DWI can be best seen in the reduction of the T2 shine-through.

**Figure 3:**
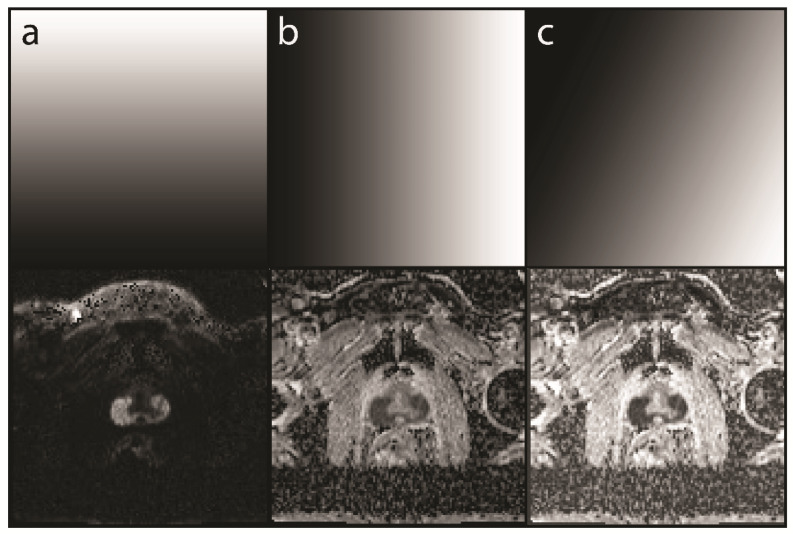
Examples of different readouts from a mcDI dataset created from a high b-value DWI (hbDWI), the ADC, and an intensity map. The selected map is shown in the top row and the resulting mcDI in the bottom row. (**a**) A readout with a strict vertical gradient yields the original DWI. The resulting image contains no ADC information; (**b**) In contrast to (**a**), a strict horizontal gradient yields the original ADC image; (**c**) A tilted gradient yields the mcDI containing both the DWI and ADC information with an even lower signal in the large tumor in the peripheral zone of the prostate and a lighter background.

The 2D histograms of these 70 patients (training dataset) were analyzed, and the PCa and normal prostate tissue areas were defined. The size, shape, and location of these areas were used to generate rules for the creation of a so-called intensity map. These rules were translated into gradients that were plotted onto the intensity map. Those rules are explained in detail in the Results. Finally, for each DWI-ADC image pair, a mcDI was generated by plotting the intensity map values back onto the initial voxel locations. For example, tumor voxel A (0.3, 0.2) corresponds to an intensity of 70% gray on the intensity map. Therefore, the voxel in the mcDI will be represented by this dark color. This whole algorithm is visualized in Figure 2. Examples of different readouts are presented in Figure 3.

### 2.7. Evaluation

Three board-certified radiologists performed three separate reads of 56 patients (the test dataset) twice. They were aware that they were evaluating patients with prostate cancer only in the peripheral zone and that there was a control group without cancer; no further clinical information was provided. The reads were itemized into three groups, including a side-by-side display, of:

- T2w, DWI, and ADC (group 1);
- T2w and mcDI (group 2);
- T2w, mcDI, DWI, and ADC (group 3).

**Figure 4:**
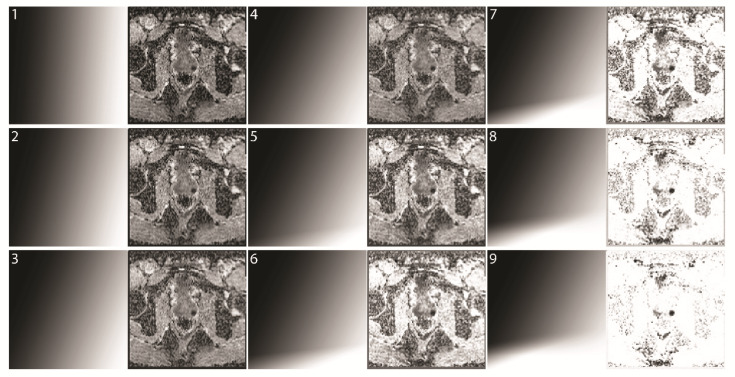
Increasing strengths of background suppression. Examples of different readouts from a mcDI dataset created from hbDWI and the corresponding ADC (map **1**–**9**). The selected map is shown on the left and the resulting mcDI on the right. Map 1 has a strict vertical readout and therefore yields the original ADC image. In the following maps, the gradient is tilted clockwise in increments to include the DWI value. Additionally, another gradient is added at the bottom to suppress the background.

Group 1 serves as the baseline performance of the readers. Although only clearly negative (PI-RADS 1 or 2) and positive (PI-RADS 4 or 5) examinations with the corresponding biopsy results were included, we expected an accuracy of less than 100% due to the mono-planar approach, missing clinical information, and nonclinical setting of the study. The readers had to decide whether a significant PCa was present. To eliminate the risk of increased accuracy in one of the groups due to a training effect, the patients and three groups were distributed randomly, each read was at least one week apart, and the results of the previous reads were not known to the observers. The second evaluation was done in the same manner. All observers received ten training patients (that were not part of the test dataset), their PI-RADS score, and histology. The reading time for each patient was recorded. The readers could select from nine different maps and the corresponding mcDI via a sliding scale (Figure 4). Map 1 was the original ADC image. In each subsequent map, the influence of the DWI and background suppression was increased.

### 2.8. Statistical Analysis

Quantitative variables were expressed as the mean ± standard deviation, whereas the categorical variables were expressed as frequencies. The sensitivity, specificity, positive predictive values (PPV), negative predictive values (NPV), and accuracy were calculated for each group, reader, and read. The null hypothesis was defined as no significant differences between the performance/time requirement of the distinct groups. A diagnostic performance was evaluated using McNemar’s test for paired nominal data. The time requirement was compared using one-way ANOVA. *p*-values were adjusted for multiple comparisons by Bonferroni correction. Intra- and inter-reader agreements were evaluated using Krippendorff’s alpha [12]. SPSS^®^ Statistics 21 (IBM Corporation, Armonk, NY, USA) and Python were used for the analysis. A *p*-value < 0.05 was considered statistically significant. Artworks were generated using SPSS^®^ Statistics 21, Adobe Photoshop, and Adobe Illustrator (both version CC, Adobe Systems, San Jose, CA, USA).

## 3. Results

The highest intensity within the DWIs (b = 0) and the ADCs corresponded with urine in the bladder. At least some amount of fluid was present in all patients, allowing us to normalize all images sufficiently.

The analysis of the histograms showed that there is a large overlap in intensity between the PCa and the healthy parenchyma. However, almost no PCa voxel demonstrated a normalized DWI of less than 0.05. This was used to implement a “no-tumor zone” in the intensity maps. Furthermore, the PCa voxels demonstrated a higher normalized DWI value in combination with a lower normalized ADC value. This is in accordance with the clinical knowledge and was therefore also implemented in the intensity maps. The steps of the intensity map creation are part of the algorithm in Figure 2.

The readers were able to select from different maps (Figure 4). They preferred intermediate background suppression. Readers 1 and 3 preferred map 5 (number of reads with the corresponding map (m) for readers 1;3: m1 = 2;0, m2 = 5;11, m3 = 9;8, m4 = 13;16, m5 = 62;63, m6 = 19;8, m7 = 1;6, m8 = 0;0, and m9 = 1;0), while reader 2 preferred a more conservative approach, with m3–m5 being his preferred maps (m1 = 0, m2 = 1, m3 = 33, m4 = 38, m5 = 37, m6 = 2, m7 = 0, m8 = 0, and m9 = 0).

**Figure 5:**
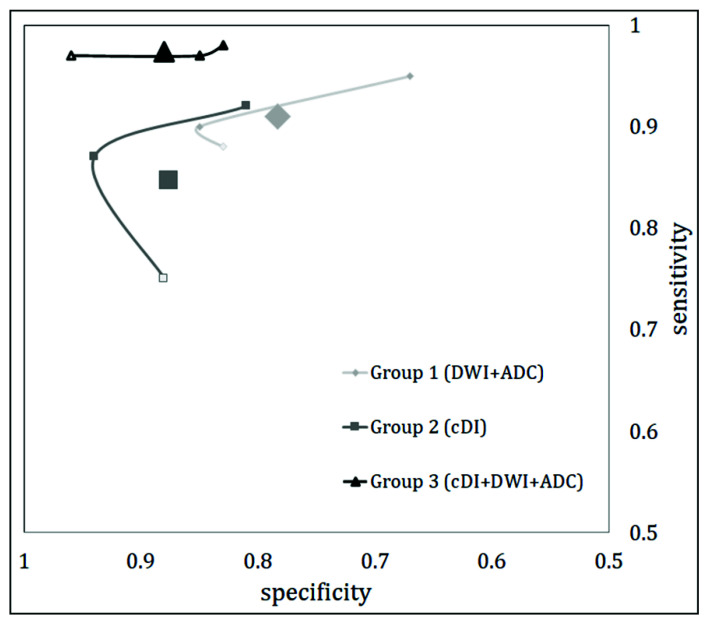
Diagnostic accuracy of the three distinct groups. The sensitivity (*y*-axis) and specificity (*x*-axis) for the three readers are plotted for the reading DWI (light gray diamond), mcDI (dark gray square), and the combined image information (black triangle). The results of the three readers are connected for visibility. The larger icon represents the mean values for all readers. Reader 1 (transparent icons) had the lowest sensitivity for identifying PCa from DWI and mcDI separately. However, his sensitivity and specificity were most improved from the combination of the two sequences. Reader 2 (middle icon) improved his specificity for reading the mcDI; however, his sensitivity decreased slightly. The combination of the DWI and mcDI improved his sensitivity without losing the specificity. Reader 3 (dark outer icon) had the highest sensitivity in all his reads. He improved his sensitivity and specificity with the combination of the two sequences.

The use of mcDI alone led to an increase in the specificity combined with a decrease in the sensitivity. However, the combined use of DWI, ADC, and the mcDI improved the overall sensitivity and specificity compared to DWI/ADC alone. The adjusted *p*-values for all comparisons were <0.001. This is described in detail in Figure 5.

The inter- and intrareader agreement are displayed in Table 1. Except in the case of reader 2, the agreement increased from group 1 to group 2 and from group 2 to group 3.

The reading times differed slightly between the distinct groups. For group 1 (DWI + ADC), the readers needed an average of 24 ± 17 s; for group 2 (mcDI), 23 ± 15 s; and for group 3 (mcDI + DWI + ADC), 28 ± 20 s (*p* < 0.001). In the post hoc pairwise *t*-tests, there was no significant difference between groups 1 and 2 (*p* = 0.974). There was, however, a significant difference between groups 1 and 3 (*p* = 0.027) and between groups 2 and 3 (*p* < 0.001).

## 4. Discussion

We created mcDI by merging the information from hbDWI and ADC into one image. It was shown that the mcDI images were not inferior to the conventional combination of the hbDWI and ADC and that mcDI can improve the sensitivity and specificity of PCa detection.

In our study, the readers could select from various maps with different weightings of the suspicious tissue and the background. The readers were found to favor an intermediate weighting of the images. One explanation for this lies in the loss of the background information necessary to correlate the tumor with its location in the prostate due to the strong suppression of the background in a mcDI setting.

In routine clinical practice, a combination of a low and a high b-value is seldom read in isolation. It is standard to calculate an ADC, and in many cases, a higher b-value is calculated to enhance the sensitivity for tumor detection [13,14]. These calculations are, however, purely mathematical and highly standardized; therefore, no knowledge of the underlying tissue is used to enhance the images, especially for a specific study. The present study produced two findings that are commonly known to radiologists but are not yet implemented in image creation. First, tumor conspicuity increases with the increasing signal in hbDWI and decreasing intensity in ADC. We used this characteristic by tilting the axis of the grayscale gradient to combine the information of both images (Figure 3). Second, most of the background has a low signal in the hbDWI, while there are no tumor voxels below a certain threshold. This no-tumor zone was used to reduce the background without losing contrast in the tumor (Figure 4).

Several studies have focused on improving the DWI and ADC. Sprinkart et al. proposed an alternative approach to generating cDWIs and altered ADCs [15]. They acquired DWIs with b-values of 0, 50, and 800 s/mm^2^ and computed DWIs with b-values of 2000 and 3000 s/mm^2^. Furthermore, they created exponential ADCs by normalizing the DWI to the signal intensity at b = 0 s/mm^2^. They found that the computed images were superior to the original DWI. We extended this approach by combining the contrasts from the ADCs and DWIs.

Kitamura et al. used an approach similar to ours for merging the information from the DWI and ADC into a single image by using 2D coordinates [5]. Again, they used a different approach for their images compared to our study. First, they did not attempt to enhance a grayscale image but used color to differentiate between T2 shine-through, restricted/facilitated diffusion, and blackout in MRI of the brain. Second, they used a mathematical approach. Compared to the study by Sprinkart et al., their approach was more complex. Instead of applying one mathematical operation to the whole image, they had four different formulas for values below and above the cut-off values of the DWI and ADC. Kitamura et al. focused on brain data, and while we preliminarily modeled their color scheme, we found it difficult to generate satisfactory prostate images. Their proposed strict cut-offs may be more suited to brain imaging. Therefore, we implemented the use of a map with smooth gradients to create high-contrast images, which are less influenced by image noise.

Our study faces limitations that suggest directions for future work. The most important limitation is that no whole amount histology of the prostate was available, as a prostatectomy is rarely performed in our hospital. Additionally, our patient population was limited, with only 56 (+70) patients included in this study. As a result, a recall bias could have been introduced that would influence the results. To compensate for this, there was a minimum interval of one week between two reads, and the patient order was changed for each reading. Another limitation of the study was that the window (W) and center (C) of the mcDI were not adjustable by the reader, which was criticized by one reader.

**Figure 6:**
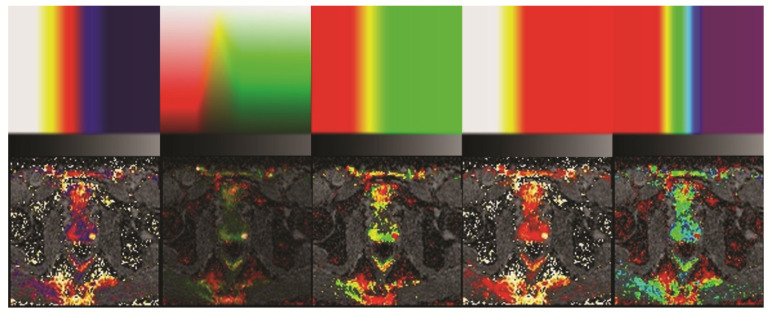
Example of different potential color maps. The map is shown in the top row and the corresponding image in the bottom row. The map is divided into two sections. The high DWI values are color-coded with different maps to better visualize the tumor (left posterior peripheral zone). The low DWI values represent the background (‘no-tumor zone’) and contain a vertical grayscale gradient. It can be noted that the highest ADC value is not white but 50% gray. This darkens the background.

Initially, the aim of this study was to create Positron Emission Tomography Computed Tomography (PET-CT)-like images using colors to highlight cancer and grayscale in the background (Figure 6). The advantages are manifold. Three color channels (red, green, and blue) can be used to convey information; theoretically, the information from three different sequences, including T2w and DCE, can be used to display the data. However, it is difficult to create visually pleasing maps, and in some cases, the tumor is even masked compared to the original image. Therefore, we discarded color maps and attempted only to improve the contrast between tumor and nontumor tissue. However, future studies can focus on color mapping. DWIs are not only used in MRIs of the prostate. Further studies may be performed to assess the mcDI maps on other organs.

## 5. Conclusions

In conclusion, we developed an algorithm that combines the information from both the hbDWI and ADC into one single mcDI. The evaluation of prostate MRI using the mcDI improved the detection of prostate cancer in the peripheral zone.

## Figures and Tables

**Table 1:** Inter- and inter-reader agreement ^1^.

Intrareader	Group 1 (DWI + ADC)	Group 2 (mcDI)	Group 3 (mcDI + DWI + ADC)
Reader 1	0.886	0.913	0.937
Reader 2	0.942	0.910	0.876
Reader 3	0.943	0.973	1.000
Inter-reader	0.733	0.800	0.853

^1^ Krippendorff’s alpha was used to calculate the intra- and inter-reader agreement.

## Data Availability

The data presented in this study are available on request from the corresponding author.
